# hUC-MSCs loaded collagen scaffold for refractory thin endometrium caused by Asherman syndrome: a double-blind randomized controlled trial

**DOI:** 10.1093/stcltm/szaf011

**Published:** 2025-05-15

**Authors:** Zhaojuan Hou, Tianli Yang, Dabao Xu, Jing Fu, Hongying Tang, Jing Zhao, Qiong Zhang, Jingjing Chen, Qun Qin, Waixing Li, Haixu Chen, Hui Li, Lei Guo, Bin Xu, Yanping Li

**Affiliations:** Center for Reproductive Medicine, Xiangya Hospital of Central South University, Changsha 410000, People’s Republic of China; the Clinical Research Center For Women’s Reproductive Health in Hunan Province, Changsha 410000, People’s Republic of China; Center for Reproductive Medicine, Xiangya Hospital of Central South University, Changsha 410000, People’s Republic of China; the Clinical Research Center For Women’s Reproductive Health in Hunan Province, Changsha 410000, People’s Republic of China; the Department of Gynecology, Third Xiangya Hospital of Central South University, Changsha 410000, People’s Republic of China; Center for Reproductive Medicine, Xiangya Hospital of Central South University, Changsha 410000, People’s Republic of China; the Clinical Research Center For Women’s Reproductive Health in Hunan Province, Changsha 410000, People’s Republic of China; Center for Reproductive Medicine, Xiangya Hospital of Central South University, Changsha 410000, People’s Republic of China; the Clinical Research Center For Women’s Reproductive Health in Hunan Province, Changsha 410000, People’s Republic of China; Center for Reproductive Medicine, Xiangya Hospital of Central South University, Changsha 410000, People’s Republic of China; the Clinical Research Center For Women’s Reproductive Health in Hunan Province, Changsha 410000, People’s Republic of China; Center for Reproductive Medicine, Xiangya Hospital of Central South University, Changsha 410000, People’s Republic of China; the Clinical Research Center For Women’s Reproductive Health in Hunan Province, Changsha 410000, People’s Republic of China; Center for Reproductive Medicine, Xiangya Hospital of Central South University, Changsha 410000, People’s Republic of China; the Clinical Research Center For Women’s Reproductive Health in Hunan Province, Changsha 410000, People’s Republic of China; the Center for Clinical Trial, Xiangya Hospital of Central South University, Changsha 410000, People’s Republic of China; the Department of Gynecology, Third Xiangya Hospital of Central South University, Changsha 410000, People’s Republic of China; the Institute of Geriatrics & National Clinical Research Center of Geriatrics Disease, the Second Medical Center, Chinese PLA General Hospital, Beijing 100000, People’s Republic of China; Center for Reproductive Medicine, Xiangya Hospital of Central South University, Changsha 410000, People’s Republic of China; the Clinical Research Center For Women’s Reproductive Health in Hunan Province, Changsha 410000, People’s Republic of China; Department of Hepatobiliary & Pancreatic Surgery, the Third Bethune Hospital of JiLin University, Changchun 130000, People’s Republic of China; Center for Reproductive Medicine, Xiangya Hospital of Central South University, Changsha 410000, People’s Republic of China; the Clinical Research Center For Women’s Reproductive Health in Hunan Province, Changsha 410000, People’s Republic of China; Center for Reproductive Medicine, Xiangya Hospital of Central South University, Changsha 410000, People’s Republic of China; the Clinical Research Center For Women’s Reproductive Health in Hunan Province, Changsha 410000, People’s Republic of China

**Keywords:** Asherman syndrome, clinical trial, intrauterine adhesion, mesenchymal stem cell, thin endometrium

## Abstract

In this single-center, double-blinded, randomized controlled trial, we investigated whether human umbilical cord-derived mesenchymal stromal cells loaded collagen scaffolds (hUC-MSC/CS) could improve the cumulative live-birth rate (cLBR) in infertile women with refractory thin endometrium (RTE). We randomly assigned 25 subfertile women with RTE, in a 1:1 ratio, to receive hysteroscopic adhesiolysis and plowing plus either hUC-MSC/CS or saline/CS (control) for intrauterine implantation. Uterine fluid was collected on the embryo transfer day for RNA-sequencing to explore the potential mechanisms by which hUC-MSCs exert their effects. The primary outcome was the cLBR. Live births occurred in 3 out of 11 women in the hUC-MSC/CS group and in 1 out of 13 women in the control group (27.3% vs 7.7%; relative risk [RR], 3.55; 95% confidence interval [CI], 0.43 to 29.42; *P* = .30). The cumulative frequencies of clinical pregnancy were 5/11 and 1/13 in the hUC-MSC/CS group and control group, respectively (45.5% vs. 7.7%; RR, 5.91; 95% CI, 0.81-43.28; *P* = .06). Two of 11 participants developed urticaria in the hUC-MSC/CS group. Enrichment analysis showed that T-cell activation had the largest proportion in the biological process category. Kyoto Encyclopedia of Genes and Genomes pathway analysis showed that most genes were related to cytokine-cytokine receptor interaction. In conclusion, there was a non-significant trend toward a higher cLBR with hUC-MSC/CS compared to controls, potentially through the cytokine-cytokine receptor interaction pathway. hUC-MSCs appeared to be relatively safe in a 1-year follow-up. Therefore, this novel therapy can be proposed to patients with RTE.

## Graphical Abstract

**Graphical Abstract:**
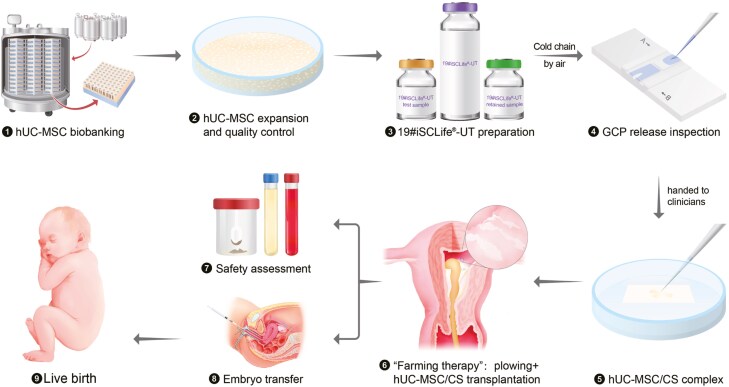

**Significance statement**

This study was a double-blinded, randomized, controlled trial of human umbilical cord mesenchymal stromal cells-seeded collagen scaffold (hUC-MSC/CS) treatment in subfertile patients with refractory thin endometrium (RTE). This study demonstrated a non-significant higher cumulative live-birth rate in the hUC-MSC/CS group compared with controls. Regarding safety issues, a portion of patients developed urticaria in the treatment group. The particularity of our study was that scratches were made by hysteroscopic plowing technique before cell transplantation. Though the study’s findings are not conclusive, the results warrant additional investigation into cell therapy for patients with RTE.

**Lessons learned**

- This double blind randomized controlled trial in subfertile subjects with refractory thin endometrium demonstrated a non-significant trend towards higher cumulative live birth rates in the hUC-MSC/CS group compared with controls.
- Two out of 11 patients experienced urticaria in the hUC-MSC/CS group.
- hUC-MSCs appeared to be relatively safe in a 1-year follow-up.
- hUC-MSCs may function through cytokine-cytokine receptor interaction pathways.
- The observed findings warrant further investigation into cell therapy for patients with refractory thin endometrium.

## Introduction

Currently, there is no consensus on the definition of a thin endometrium; however, it is generally considered to be less than 7 mm in thickness during the late proliferative phase.^1,2^ The prevalence of thin endometrium in assisted reproductive technology (ART) varies between 2.40% and 5.45%.^3,4^ Intrauterine adhesion (IUA), also known as Asherman syndrome (AS), is the leading cause of endometrial thinness.^5^ Refractory thin endometrium (RTE) is characterized by persistently thin lining (<7 mm) despite standard combined treatment of hysteroscopic adhesiolysis and hormone replacement therapy (HRT).

Over the past 3 decades, heterogeneous strategies have been developed to address a thin endometrial lining, including prolonged estrogen administration,^6^ low-dose aspirin,^7^ intrauterine injection of granulocyte colony-stimulating factor^8^ or platelet-rich plasma,^9^ vaginal sildenafil suppositories,^10^ hyperbaric oxygen therapy,^11^ and integrative treatment of pentoxifylline and tocopherol.^12^ Despite these interventions, some patients remain unresponsive, leading to repeated cycle cancelations during ART treatment, embryo implantation failure, and recurrent pregnancy loss.

In recent years, stem cell therapy has emerged as an attractive alternative against intractable diseases and injuries due to their infinite proliferation and pluripotency.^13^ Santamaria et al reported a sophisticated catheterization for delivering autologous CD133 + bone marrow-derived stem cells (BMDSCs) into the spiral arterioles to treat endometrial atrophy.^14^ Others attempted to repair the traumatized endometrium by direct infusion of human umbilical cord-derived mesenchymal stromal cells (hUC-MSCs) into the uterine cavity.^15^ Additionally, 2 studies reported hUC-MSCs loaded on a collagen scaffold (CS) for treating RTE or recurrent AS.^5,16^ With respect to clinical application, one issue needs to be further refined: the route of transplantation.

To investigate the efficacy and safety of a novel approach for hUC-MSCs transplantation, we performed a single-center, double-blinded, randomized controlled trial (RCT) via hysteroscopic adhesiolysis and plowing, followed by transplantation of hUC-MSCs or saline-loaded CS into the uterine cavity. Putative mechanisms were further explored by RNA sequencing (RNA-seq) and data mining of uterine fluid samples from study participants.

## Methods

### Trial design and oversight

**Figure 1.**
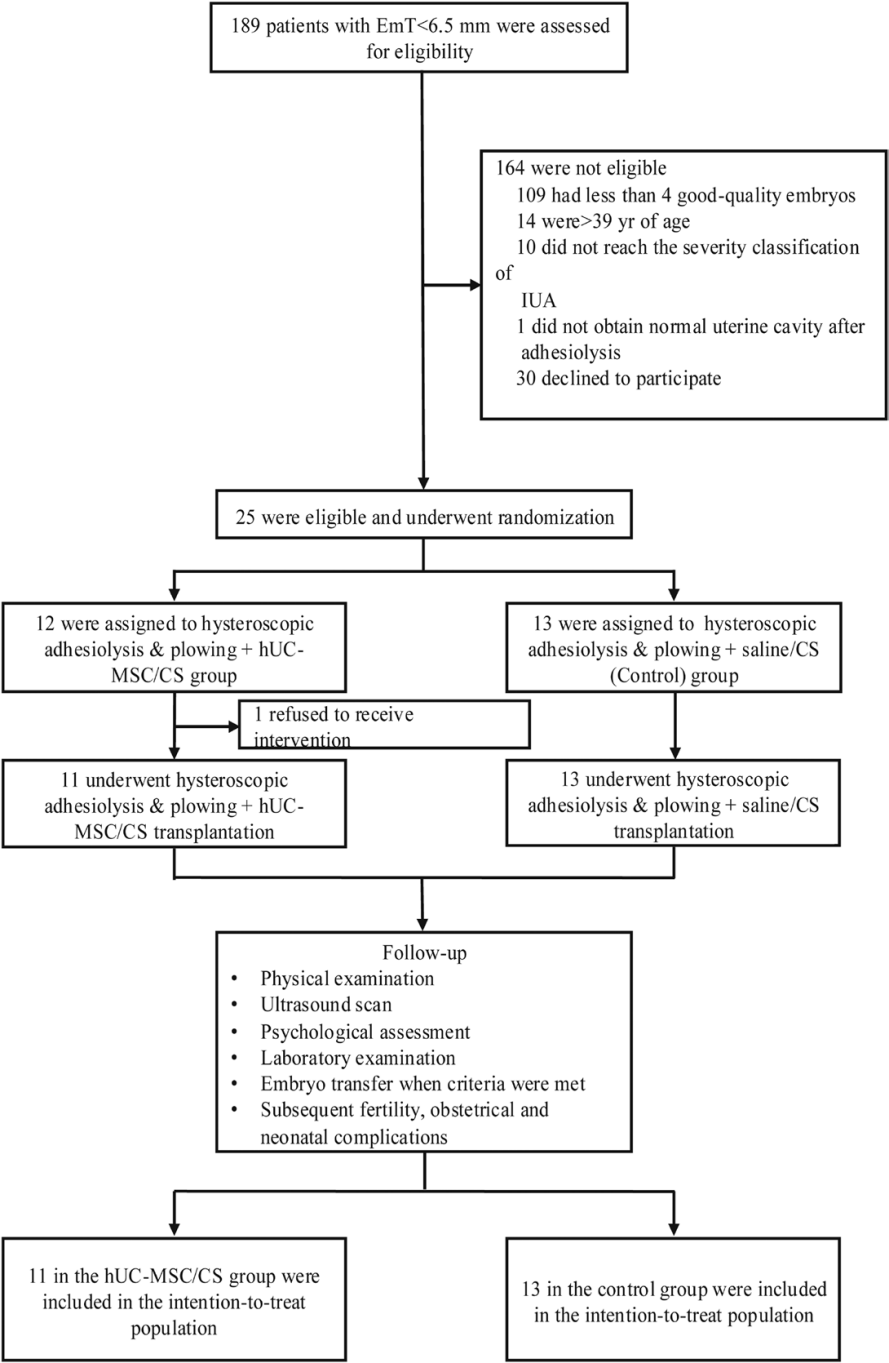
CONSORT flow diagram.

This clinical trial was a single-center, double-blinded RCT designed to evaluate the efficacy and safety of combining hysteroscopic adhesiolysis and plowing with hUC-MSC/CS complex transplantation for the treatment of RTE. Moreover, potential therapeutic mechanisms of hUC-MSCs were explored through RNA-seq and bioinformatics analysis. The Consolidated Standards of Reporting Trials flow diagram is illustrated in Figure 1.

### Study approval

This trial was approved by the Ethics and Academic Committee of Stem Cell Clinical Research Institution of Xiangya Hospital, Central South University in China (No. 201901001). The study was conducted at the Reproductive Medicine Center of Xiangya Hospital from August 2022 to November 2023 and was registered on ClinicalTrials.gov (registration number: NCT05495711). All participants received comprehensive counseling regarding the risks and benefits of the study, and informed consent was obtained before their participation and sample collection.

### Patients

Eligible women were 20-39 years old with a body mass index ranging from 18 to 24 kg/m^2^, had been diagnosed with infertility, and had received ART with at least 4 good-quality embryos stored to ensure 2 rounds of embryo transfer (ET). Blastocysts were deemed to be good if the score on day 5 of culture was 4BC or higher, according to the evaluation system proposed by Gardner.^17^ Cleavage embryos were classified as good quality if they were graded I or II with fragmentation <5% based on the Istanbul Consensus.^18^ The severity of IUA was classified as Grade III or higher (according to the European Society of Gynecological Endoscopy, 1995)^19^ or as stage III (Severe) based on the American Fertility Society scoring system (AFS, 1988).^20^ Furthermore, transcervical resections of adhesion were carried out until no fibrous strips existed in the uterine cavity, and the AFS score was below 4. Additional eligibility criteria included a consistently thin endometrial thickness (EmT) <6.5 mm after the administration of at least 8 mg/day estradiol valerate for over 12 days post-adhesiolysis, along with voluntary participation and provision of informed consent.

Women were excluded if they had untreated conditions that were associated with adverse pregnancy outcomes (eg, hydrosalpinx, endometrial polyps, intrauterine infection, stage III-IV endometriosis, adenomyosis, ovarian cyst or benign abdominopelvic tumors with a diameter >4 cm, myomas with a diameter >2 cm, multiple uterine fibroids, intramural or submucous myomas, pituitary tumors, or malignant tumors). Additional exclusion criteria are detailed in the Supplementary Appendix.

### Randomization and masking

A random sequence was generated using IBM SPSS (IBM, USA, version 26.0) with a predefined random seed by a researcher at Xiangya Hospital. Patients were then randomly assigned (1:1) to the hysteroscopic adhesiolysis and plowing combined with either hUC-MSC/CS group or saline/CS (control) group on the first day of menstruation after screening.

Blinding of treatments was not feasible due to the visual difference between the cell suspension and saline solution. To maintain a double-blind study design, the interventions were performed by unmasked surgeons, while a reproductive doctor assessing EmT and participants remained blinded. The reproductive doctor who measured EmT in subsequent frozen embryo transfer (FET) cycles was unaware of patient data and the specific intervention each patient received. Surgeons were prohibited from sharing information about the intervention with patients and were not involved in any clinical assessments of the endometrium.

### Manufacture of clinical-grade hUC-MSCs

The clinical-grade hUC-MSCs were prepared in a Good Manufacturing Practice (GMP) laboratory as previously reported.^15^ Furthermore, the quality of hUC-MSCs was tested and certified by the National Institutes for Food and Drug Control of China on September 4, 2017. The hUC-MSCs were sourced from a single healthy donor at term delivery who signed the informed consent. The donor was free of hepatitis A, B, and C viruses, human immunodeficiency virus, Epstein-Barr virus, cytomegalovirus, treponema pallidum, mycoplasma, and chlamydia as confirmed by the donor’s attending physician prior to cord collection.

During the isolation process, the Wharton’s jelly was mechanically separated from the blood vessels. Then tissues were diced into small pieces (1 mm^3^) and subjected to an optimized enzymatic digestion, followed by culturing with Alpha-Minimum Essential Medium (α-MEM) containing 5% UltraGRO-Advanced (AventaCell) at 37°C in 5% CO_2_. Next, the culture medium was refreshed every 3 days until passage. Detached cells were centrifuged, washed, and passaged for expansion in α-MEM (Corning) supplemented with 5% UltraGRO-Advanced (AventaCell) at 37°C in 5% CO_2_. For cryopreservation, the cells were detached and centrifuged. Subsequently, they were resuspended in cryopreservatives at a concentration of (1.7 ± 0.34) × 10^6^ cells/mL in a volume of 0.8 mL/vial and transferred to liquid nitrogen for long-term storage.

The hUC-MSCs were thawed and cultured and then formulated as cell products at the fifth passage, which should be kept at 2-8°C for clinical use. The clinical-grade hUC-MSCs preparations were produced by resuspending 2 × 10^7^ hUC-MSCs with cell-preserving material iECM to a total volume of 2 mL. The iECM was prepared as a solution at a concentration of 1 mg/mL. The iECM was procured from donated Wharton’s jelly under GMP conditions. First, the Wharton’s jelly was homogenized and centrifuged with the supernatant was collected. Afterward, decellularized iECM was obtained by centrifugation at 3000 g for 30 minute The ECM was mainly composed of collagen and glycosaminoglycans, which was subjected to sterile testing. The release specification before shipment included confirmation of cell count ([2 ± 0.2] × 10^7^), cell viability (≥90%), identity (CD73, CD105, CD29, CD90, CD276, CD146, and HLA-ABC ≥ 95%), purity (CD34, CD45 and HLA-DR ≤ 2%), potency (adipogenic and osteogenic potential in vitro), sterility (bacteria and fungi), and absence of endotoxin and mycoplasma contaminations. In addition, a host of biological factors, including but not limited to hepatocyte growth factor (R&D), insulin-like growth factor (R&D), fibroblast growth factor (R&D), and epidermal growth factor (R&D), were tested. Furthermore, the hUC-MSCs exhibited inhibitory effects in T-cell proliferation assay in vitro (details on hUC-MSCs characterization and the suppliers of antibodies and differentiation kit are provided in Supplementary Figure S1 and Table S1 in the Supplementary Appendix, respectively). The cells were shipped to the research site under temperature-controlled conditions at 2-8°C. Before coating onto the CS for intrauterine implantation, these preparations were re-examined by the officers at the Center for Clinical Trial of Xiangya Hospital, based on the Release Inspection Guideline (cell count should be within ± 10% of the specified count before shipment, with a viability ≥ 80%).

### Interventions and procedures

Prior to hUC-MSC/CS or saline/CS transplantation, hysteroscopic adhesiolysis and plowing were performed simultaneously under hysteroscopy. The plowing technique involved creating several longitudinal incisions in the scar tissue to form narrow strips. Here, hysteroscopic plowing coupled with hUC-MSC/CS transplantation was termed “farming therapy,” which facilitated the adhesion of stem cells to the endometrium, allowing them to remain for a longer period of time.^21^ The operation was carried out by Professor Xu at the Third Xiangya Hospital of Central South University on the 10^th^ to 12^th^ day of the menstrual cycle.

The fabrication of hUC-MSCs or saline with CS was performed within 2 hours after Good Clinical Practice release inspection. A suspension of hUC-MSCs at a concentration of 1 × 10^7^/mL (2 mL) or 2 mL of saline was evenly dripped onto a CS (Zhenghai Biotechnology Co., Shandong, China). The CS was a xenogenic acellular dermal matrix prepared from bovine skin tissue, which was mainly composed of collagen. Previous studies have shown that it can degrade within one month in the uterine cavity.^5^ The CS was 4 × 3 cm^2^ with pore diameters ranging from 20 to 200 μm. It should be thoroughly soaked in sterile saline before use, and applied to the defect tissue with the “UP” side facing outward. The seeded scaffold was then incubated in a humidified incubator with 5% CO_2_ in air at 37°C for 1 hour before transplantation.

The patients were randomly assigned into 2 groups: the hUC-MSC/CS group or the control group. First, the residual liquid (either cell suspension or saline) in the culture dish was administered into the uterine cavity through an intrauterine insemination catheter. The CS complex was spread over an 8F Foley catheter and placed into the uterine lumen under ultrasound guidance. After that, the catheter bulb was filled with 2.5 mL of sterile saline to attach the scaffold complex against the inner wall of the endometrial cavity. Cell transplantation was implemented by 2 experienced doctors at Xiangya Hospital following hysteroscopic adhesiolysis and plowing. Antibiotics were used for 3 days to prevent infection, and the balloon was removed after 3 days. All subjects received 6 mg/day estradiol valerate (Progynova, Delpharm Lille) from the third day of menstruation to the 20^th^ day post-surgery. Starting from the 15^th^ day post-surgery, 200 mg of progesterone capsules (Qining; Aisheng, China) were taken orally. HRT was then discontinued, and subjects were invited to undergo HRT for FET cycles. HRT was administrated in a stepwise manner: estradiol valerate was initiated at 6 mg/day on the third day of the menstrual cycle for 6 days, and then the dosage was increased to 8 mg/day for an addtional 6 days. When the endometrium on ultrasound presented any of the following signs: EmT ≥ 6.5 mm, or an increase of >2 mm in EmT or an improvement in endometrial pattern (EmP) with a triple-layer pattern (pattern A or B), luteal-phase support with vaginal progesterone capsules (Utrogestan, Besins-Iscovesco Pharmaceuticals, France) at 200 mg 3 times daily and oral progesterone soft capsules at 200 mg daily was initiated and continued until the day of serum human chorionic gonadotropin testing. Up to 2 cleavage embryos or blastocysts were thawed and transferred 3 or 5 days, respectively, after the initiation of progesterone. Investigators had the option to cancel cycles if none of the predefined criteria mentioned were met, resulting in 29 out of 64 cycles being canceled. Endometrial aspiration was performed as previously described^22^ and uterine fluid samples were collected on the ET day for RNA-seq analysis.

### Follow-up

Physical examination, 2D ultrasound scan (EmT and EmP), and psychological assessment were performed at baseline and at 1 month follow-up. A 3D ultrasound scan (to reconstruct the shape of the uterine cavity), laboratory examinations, and an electrocardiogram (ECG) were conducted at baseline and at the 3-month follow-up. Follow-up ended with delivery after conception or one-year post-therapy for those who did not conceive. Pregnancy outcomes, along with obstetrical and perinatal complications, were meticulously documented by reviewing maternal and infant medical records.

The uterus with the cervical canal was displayed in a sagittal plane on ultrasound for EmT and EmP measurements. Measurements were obtained using an 8-MHz transduser (GE Voluson S6, USA) by Dr. Y.L, and each value was assessed as the mean of 3 consecutive measurements. 3D ultrasonography was performed by an expert examiner and analyzed by 2 specialists. Mental health variables were evaluated using the Symptom Check List (SCL-90).

### Outcomes

The primary outcome was the cumulative live birth rate (cLBR), defined as the total viable deliveries of at least 28 weeks of gestation during the follow-up divided by the total number of participants in each group. Secondary efficacy outcomes included the rates of cumulative biochemical pregnancy, cumulative clinical pregnancy, implantation, cumulative ongoing pregnancy, early spontaneous abortion, ectopic pregnancy, and cycle cancelation, as well as measurements of EmT, EmP, and SCL-90 scores. Safety outcomes were evaluated through laboratory tests and ECGs. Laboratory examinations included a complete blood count, urinalysis, routine stool tests, hepatic and renal function tests, coagulation analysis, tumor biomarkers, serum glucose and lipid levels, serum ion level, thyroid function tests, and blood transfusion laboratory tests. In addition, obstetrical and perinatal complications were recorded as part of the safety outcomes. Criteria for secondary efficacy and safety outcomes are detailed in Table S2 in the Supplementary Appendix. Serious adverse events refer to medical events that: 1. were fatal or life-threatening; 2. required unplanned or prolonged hospitalization; 3. resulted in permanent or apparent disability; 4. caused lasting damage to organ function; 5. involved congenital malformation or birth defect; 6. were otherwise medically significant events requiring medical intervention to prevent the above events.

### Transcriptome sequencing and data processing

Total RNA was extracted from the uterine fluid using the RaPure Total RNA Micro Kit & DNase Set (Magen). The concentration of the extracted RNA was quantified with the Qubit RNA HS Kit (Thermo Fisher Scientific, Waltham). Subsequently, the RNA samples were reverse transcribed into cDNA utilizing the MALBAC Platinum Single Cell RNA Amplification Kit (Yikon Genomics). The cDNA products were purified using the DNA Clean & Concentrator Kit (Zymo). Concentration measurements were performed again with the Qubit HS DNA Kit, and samples with concentrations of ≥ 1 ng/μL were considered eligible for library preparation. Following this, the samples underwent DNA fragmentation, end repair, adapter ligation, and PCR enrichment purification to complete the library construction. The constructed libraries were quantified using the Qubit dsDNA HS Assay Kit (Thermo Fisher Scientific) and sequenced on the HiSeq 2500 platform (Illumina).

We analyzed transcriptomic data between the hUC-MSC/CS group (*n* = 5) and the control group (*n* = 6) on the ET day. Gene expression levels were transformed using log_2_ (Fragments Per Kilobase of exon per Million mapped fragments), and then differentially expressed genes (DEGs) were identified by Student’s *t*-test. Gene expression variability and dimensionality reduction were conducted via principal component analysis (PCA). Gene Ontology annotations and Kyoto Encyclopedia of Genes and Genomes (KEGG) pathways analyses for these DEGs were performed using the Database for Annotation, Visualization and Integrated Discovery (https://davidbioinformatics.nih.gov), with a significant threshold set at an adjusted *P*-value < .05 and log_2_(fold change) > 1. The deconvolution method was applied to estimate the proportions of different cell types from bulk RNA-seq data^23,24^ (detailed information is provided in the Supplementary Appendix).

### Statistical analysis

Assuming an absolute difference of 30% in the live birth rate between the 2 groups, with 80% power at a significance level of.05 and a 1:1 sampling ratio, the minimum required sample size was calculated to be 33 participants per group. However, owing to the lack of long-term safety monitoring for offspring obtained from women receiving stem cell transplantation, the Ethics and Academic Committee approved a final sample size of 24, prioritizing caution and offspring safety. With this sample size, the study had a post-hoc power of 57% at a 2-sided significance level of.05 to detect an absolute difference of 37.8 percentage points in the clinical pregnancy rate between the 2 groups (45.5% in the hUC-MSC/CS group vs. 7.7% in the control group).

The analysis was performed based on the intention-to-treat principle. Prior to statistical analysis, data normality was assessed using the Shapiro‒Wilk test. Continuous variables with a normal distribution were expressed as the mean ± standard deviation, with between-group differences compared by 2 independent sample *t*-test or paired Student’s *t* test; continuous variables with a non-normal distribution were described as median (interquartile range) and compared using the Mann‒Whitney *U* test or Wilcoxon signed-rank test. Categorical data were presented as frequencies and percentages, with differences tested by the chi-square test or Fisher’s exact test. Relative risk (RR) and 95% confidence interval (CI) were presented to measure the effect size of hUC-MSCs on reproductive outcomes. A 2-sided *P* value < .05 was considered statistically significant.

## Results

### Patients

A total of 189 participants were screened, of whom 25 were eligible and randomized (Figure 1). Before treatment initiation, one participant in the hUC-MSC/CS group withdrew because of personal reasons. Among the remaining 24 participants, 11 received hysteroscopic adhesiolysis and plowing followed by hUC-MSC/CS transplantation, while 13 underwent hysteroscopic adhesiolysis and plowing followed by saline/CS transplantation. The baseline characteristics of the participants were similar in the hUC-MSC/CS group and the control group (Table 1).

**Table 1.** Characteristics of the patients at baseline.

Characteristic	hUC-MSC/CS (*n* = 11)	Control (*n* = 13)	*P*
Age-yr	34.1 ± 3.2	32.8 ± 3.4	.34
BMI^a^	21.4 ± 2.1	22.8 ± 1.1	.08
Duration of infertility-yr	2.0 (1.0 to 4.0)	2.0 (1.0 to 3.5)	.91
ART etiology-no. (%) Tubal factor Male factor PGT^b^	9 (81.8) 2 (18.2) 0 (0)	9 (69.2) 1 (7.7) 3 (23.1)	.12
Duration of IUA-yr	2.5 ± 1.4	3.0 ± 1.9	.44
IUA etiology-no. (%) D&C^c^ Unknown^d^	8 (72.7) 3 (27.3)	11 (84.6) 2 (15.4)	.63
D&C times	1.0 (0 to 2.0)	2.0 (1.0 to 2.5)	.33
Maximum IUA score	10.0 (8.0 to 11.0)	10.0 (8.0 to 10.0)	.39
Prior repair attempts	4.0 (3.0 to 6.0)	5.0 (3.0 to 6.0)	.78
Pre-EmT^e^-mm	5.3 ± 0.7	5.3 ± 0.8	.95
Pre-EmP^f^ A/B C	2 (18.2) 9 (81.8)	5 (38.5) 8 (61.5)	.26
Prior failed embryo transfer cycles	0 (0 to 2.0)	1.0 (0 to 2.0)	.65
SCL-90 positive case	1 (9.1)	4 (30.8)	.33

^a^BMI, body mass index, is the weight in kilograms divided by the square of the height in meters.

^b^PGT, preimplantation genetic testing.

^c^D&C, dilatation and curettage.

^d^Unknown, 5 participants all had a history of pulmonary tuberculosis without intrauterine tuberculosis diagnosed by hysteroscopy and endometrial biopsy.

^e^Pre-EmT, endometrial thickness measured on the day progesterone was started at baseline.

^f^Pre-EmP, endometrial pattern measured on the day progesterone was started at baseline.

### Live birth and secondary outcomes

The cLBR was 27.3% (3 of 11 women) in the hUC-MSC/CS group compared to 7.7% (1 of 13 women) in the control group (RR, 3.55; 95% CI, 0.43–29.42; *P* = .30) (Table 2). The incidence of cumulative clinical pregnancy was 45.5% (5 pregnancies out of 11 women) in the hUC-MSC/CS group and 7.7% (1 pregnancy out of 13 women) in the control group (RR, 5.91; 95% CI, 0.81–43.28; *P* = 0.06). The gestational sacs obtained from pregnant patients in the hUC-MSC/CS group are shown in Supplementary Figure S2, with their clinical characteristics presented in Supplementary Table S6. There was no difference in other secondary reproductive outcomes and obstetrical and perinatal complications between the 2 groups.

**Table 2.** Primary, secondary, and safety outcomes.

Outcome	hUC-MSC/CS Group (*n* = 11)	Control Group (*n* = 13)	RR(95% CI)	*P*
Primary outcome				
Cumulative live birth-no.(%)	3 (27.3)	1 (7.7)	3.55 (0.43 to 29.42)	.30
Secondary outcomes				
Cumulative biochemical pregnancy-no.(%)	6 (54.5)	2 (15.4)	3.55 (0.89 to 14.15)	.08
Cumulative clinical pregnancy-no.(%)	5 (45.5)	1 (7.7)	5.91 (0.81 to 43.28)	.06
Implantation rate-no./total no.(%)	5/35 (14.3)	1/22 (4.5)	3.14 (0.39 to 25.15)	.47
Cumulative ongoing pregnancy-no.(%)	3 (27.3)	1 (7.7)	3.55 (0.43 to 29.42)	.30
Early spontaneous abortion-no./total no.(%)	2/5 (40.0)	0/1 (0.0)	NA	NA
Ectopic pregnancy-no./total no.(%)	0/5 (0.0)	0/1 (0.0)	NA	NA
Safety outcomes				
Gestational diabetes among clinical pregnancies-no./total no.(%)	1/5 (20.0)	0/1 (0.0)	NA	NA
Gestational hypertension among clinical pregnancies-no./total no.(%)	1/5 (20.0)	0/1 (0.0)	NA	NA
Intrahepatic cholestasis of pregnancy among clinical pregnancies-no./total no.(%)	1/5 (20.0)	0/1 (0.0)	NA	NA
Preeclampsia among clinical pregnancies-no./total no.(%)	1/5 (20.0)	0/1 (0.0)	NA	NA
Premature rupture of membrane among clinical pregnancies-no./total no.(%)	0/5 (0.0)	0/1 (0.0)	NA	NA
Preterm delivery among clinical pregnancies-no./total no.(%)	1/5 (20.0)	0/1 (0.0)	NA	NA
Placenta previa among clinical pregnancies-no./total no.(%)	0/5 (0.0)	0/1 (0.0)	NA	NA
Placenta abruption among clinical pregnancies-no./total no.(%)	0/5 (0.0)	0/1 (0.0)	NA	NA
Placenta accreta among clinical pregnancies-no./total no.(%)	1/5 (20.0)	1/1 (100.0)	0.20 (0.04 to 1.15)	0.33
Cervical incompetence among clinical pregnancies-no./total no.(%)	1/5 (20.0)	0/1 (0.0)	NA	NA
Congenital anomalies among clinical pregnancies-no./total no.(%)	0/5 (0.0)	0/1 (0.0)	NA	NA
Postpartum hemorrhage among all deliveries-no./total no.(%)	0/3 (0.0)	0/1 (0.0)	NA	NA
Puerperal infection among all deliveries-no./total no.(%)	0/3 (0.0)	0/1 (0.0)	NA	NA
Stillbirth among all deliveries-no./total no.(%)	0/3 (0.0)	0/1 (0.0)	NA	NA
Neonatal respiratory distress syndrome among live birth-no./total no.(%)	1/3 (33.3)	0/1 (0.0)	NA	NA
Neonatal jaundice among live birth-no./total no.(%)	1/3 (33.3)	0/1 (0.0)	NA	NA
Neonatal infection among live birth-no./total no.(%)	1/3 (33.3)	0/1 (0.0)	NA	NA
Neonatal death among live birth-no./total no.(%)	0/3 (0.0)	0/1 (0.0)	NA	NA

The EmT assessed on the day of starting progesterone after treatment was 6.5 ± 1.3 mm in the hUC-MSC/CS group and 6.0 ± 1.2 mm in the control group (*P* = .33) (Table 3). The change in EmT (△EmT = post-treatment EmT–pre-treatment EmT) on the day of starting progesterone was 1.2 ± 1.2 mm in the hUC-MSC/CS group and 0.6 ± 1.6 mm in the control group, respectively (*P* = .38). The EmP A/B after treatment was 54.5% and 61.5% in the hUC-MSC/CS and the control group, respectively (*P* = .53). In the hUC-MSC/CS group, the post-treatment EmT was significantly higher than the pre-treatment EmT (Table 4, 6.5 ± 1.3 mm vs. 5.3 ± 0.7 mm, *P* = .007), whereas no significant difference was observed in the control group (6.0 ± 1.2 mm vs 5.3 ± 0.8 mm, *P* = .188). These indicated that the farming therapy increased the EmT. Both groups showed a higher proportion of EmP A/B post-treatment compared to pre-treatment, though the difference was not significant (54.5% vs 18.2% in the hUC-MSC/CS group, *P* = .289; 61.5% vs 38.5% in the control group, *P* = .453). The 3D ultrasound revealed a better structured uterine cavity in the hUC-MSC/CS group post-treatment compared to the control group (Supplementary Figure S3). Representative 2D ultrasound images of the endometrium are presented in Supplementary Figure S4. The cancelation rate for FET cycles was 36.4% (12/33) in the hUC-MSC/CS group, compared to 54.8% (17/31) in the control group (*P* = 0.14), suggesting that farming therapy with hUC-MSC/CS may provide participants with more opportunities for ET. There was no difference in terms of the cumulative number of embryos transferred, embryo stage or good-quality embryo rate between the 2 groups (*P* = .052, *P* = .49, and *P* = .38, respectively). The number of SCL-90-positive cases and scores across the 10 dimensions of the SCL-90 were comparable between the 2 groups (*P* > .05) (Table 3 and Supplementary Table S3). However, in the hUC-MSC/CS group, post-treatment scores for obsessive-compulsive symptoms and phobic anxiety were significantly lower than pre-treatment scores (Supplementary Table S4). In addition, in the control group, post-treatment scores for the global severity index (GSI) and interpersonal sensitivity were significantly lower compared to pre-treatment scores. Detailed information on the ten dimensions and GSI scores of the SCL-90 is provided in Supplementary Table S3 and S4.

**Table 3.** Other secondary efficacy outcomes and embryo information.

	hUC-MSC/CS (*n* = 11)	Control (*n* = 13)	*P*
^a^Post-EmT	6.5 ± 1.3	6.0 ± 1.2	.33
^b^△EmT	1.2 ± 1.2	0.6 ± 1.6	.38
^c^Post-EmP A/B C	6 (54.5) 5 (45.5)	8 (61.5) 5 (38.5)	.53
Cycle cancelation rate	12/33 (36.4)	17/31 (54.8)	.14
No. of cumulative embryos transferred	3.0 (2.8 to 4.3)	2.0 (1.0 to 4.0)	.052
Embryo stage Day3 Day5	16/35 (45.7) 19/35 (54.3)	8/22 (36.4) 14/22 (63.6)	.49
Good-quality embryo	25/35 (71.4)	18/22 (81.8)	.38
^d^SCL-90 positive case	1 (9.1)	2 (15.4)	1.0

^a^Post-EmT, endometrial thickness on the day of starting progesterone after intervention.

^b^△EmT = post-EmT-pre-EmT, differences of endometrial thickness on the day of starting progesterone between post-intervention and baseline.

^c^Post-EmP, endometrial pattern on the day of starting progesterone after intervention.

^d^SCL-90 positive case, assessed after intervention.

**Table 4.** Comparison of pre- and post-EmT (endometrial thickness) and EmP (endometrial pattern) on the day of starting progesterone.

	*n*	EmT-mm			EmP		
		Pre	Post	*P*	Pre-A/B	Post-A/B	*P*
hUC-MSC/CS	11	5.3 ± 0.7	6.5 ± 1.3	.007	2 (18.2)	6 (54.5)	.289
Control	13	5.3 ± 0.8	6.0 ± 1.2	.188	5 (38.5)	8 (61.5)	.453
Total	24	5.3 ± 0.7	6.2 ± 1.2	.006	7 (29.2)	14 (58.3)	.118

### Safety outcomes

No SAE occurred after intrauterine transplantation of hUC-MSC/CS. Common post-infusion symptoms such as transient fever, chills, and headache, often associated with hUC-MSCs administration,^25,26^ were not observed in any participants in our trial. Some patients experienced mild dizziness, nausea, and lower abdominal pain post-procedure, which resolved within a few hours (Supplementary Table S5). Urticaria occurred in 2 out of 11 participants after the hUC-MSC/CS transplantation, which resolved within a few days without special treatment. The following parameters, including coagulation function, liver and renal function, serum ionized calcium, sodium and potassium concentrations, thyroid function, and tumor markers, showed no statistically significant differences between pre- and post-hUC-MSC/CS treatments (Table 5). Routine urine and feces tests, as well as ECG, showed no notable abnormalities throughout the 3-month follow-up period. However, significant changes were observed in platelet count, fasting blood glucose, triglycerides, serum chloride concentration, and carbohydrate antigen 72-4 levels after hUC-MSC/CS therapy, with all values remaining within the normal range except for triglycerides.

**Table 5.** Safety evaluation on patients pre- and post-hUC-MSC/CS.

Parameter	pre-hUC-MSC/CS *n* = 11	post-hUC-MSC/CS *n* = 11	P
WBC × 10^9^/L	5.8 ± 1.2	6.8 ± 1.8	.05
Hb g/L	130.2 ± 12.2	129.2 ± 11.9	.56
PLT × 10^9^/L	214.4 ± 58.6	226.9 ± 53.4	.049
RBC × 10^12^/L	4.5 ± 0.4	4.5 ± 0.5	.73
D-Dimer mg/L	0.1 (0-0.1)	0.1 (0-0.1)	.69
ALT U/L	16.6 (12.0-40.7)	23.1 (9.7-37.8)	.27
AST U/L	25.7 ± 10.5	23.9 ± 7.1	.48
BUN mmol/L	4.7 ± 1.0	4.3 ± 0.7	.27
Albumin g/L	44.2 ± 2.1	44.1 ± 2.0	.96
Creatinine ummol/L	62.0 (53.0-67.0)	59.0 (49.0-67.0)	.29
TBIL umol/L	9.2 ± 2.7	10.0 ± 2.3	.31
DBIL umol/L	2.9 (2.3-4.0)	2.8 (2.5-4.0)	.86
TBA umol/L	2.2 (1.0-3.9)	2.6 (1.2-4.4)	.33
Uric Acid umol/L	311.9 ± 71.2	315.2 ± 71.2	.85
FBG mmol/L	5.5 ± 0.4	5.1 ± 0.7	.03
TG mmol/ L	1.5 ± 0.7	1.9 ± 0.7	.01
TC mmol/L	4.8 (4.5-5.0)	4.8 (4.6-5.4)	.18
HDL-C mmol/L	1.4 ± 0.2	1.4 ± 0.3	.63
LDL-C mmol/L	2.9 (2.7-3.4)	3.0 (2.9-3.5)	.09
K + mmol/L	4.2 ± 0.2	4.0 ± 0.2	.10
Na + mmol/L	140.0 ± 1.7	139.2 ± 1.9	.10
Cl- mmol/L	104.5 ± 2.4	103.5 ± 2.1	.02
Ca2 + mmol/L	2.4 (2.3-2.4)	2.4 (2.3-2.5)	.42
TSH uIU/ml	2.1 ± 1.2	1.6 ± 0.8	.25
FT3 pmol/L	4.8 ± 0.6	4.8 ± 0.8	.84
FT4 pmol/L	16.6 ± 2.6	16.7 ± 4.1	.91
CEA ng/mL	0.7 (0.5-1.2)	0.7 (0.6-0.9)	.35
AFP ng/mL	2.6 ± 1.0	2.5 ± 1.1	.60
CA15-3 KU/L	3.7 (3.6-4.4)	3.9 (3.2-4.7)	.86
CA199 ng/mL	5.0 ± 3.1	4.8 ± 3.2	.70
CA125 U/mL	7.6 (5.2-9.8)	4.5 (4.3-9.5)	.37
CA72-4 U/m	0.8 (0.4-1.9)	0.6 (0.3-0.9)	.04
NSE ng/mL	6.5 (4.4-8.2)	4.8 (4.5-4.9)	.33
PGI ng/mL	43.8 (40.7-51.9)	44.7 (31.4-55.5)	.21
PGII ng/mL	4.3 (3.8-6.7)	4.9 (3.6-6.0)	.66
PGI/PGII	9.6 ± 4.6	8.6 ± 3.5	.39

### DEGs detection and functional analysis

**Figure 2.**
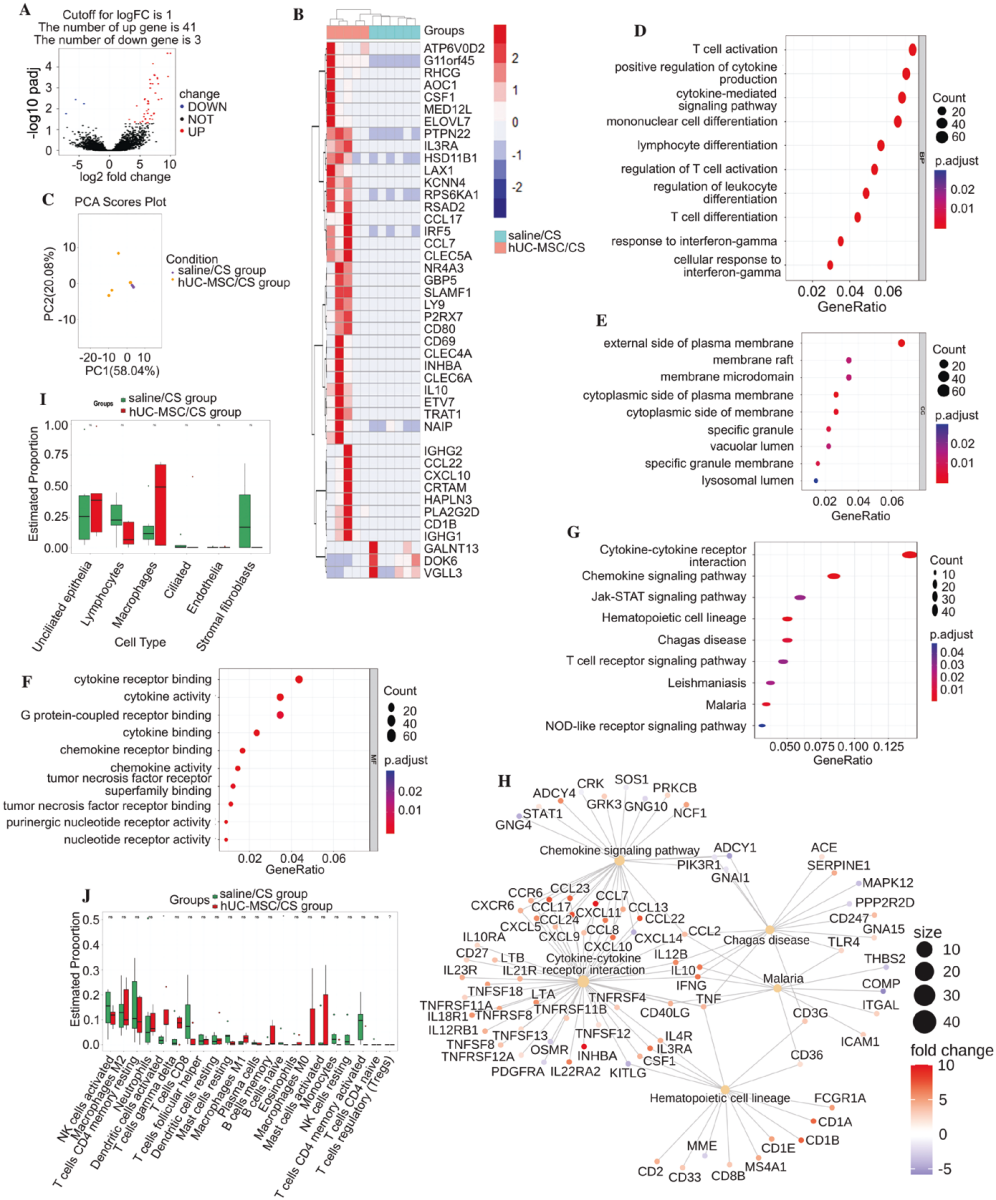
Identification and enrichment analysis of RNA-Seq differentially expressed genes (DEGs) and cell proportion analysis between hUC-MSC/CS participants and controls. Volcano plot (A) and heatmap (B) showing 41 up- and 3 downregulated DEGs (adjusted *P*-value < .05 and log_2_FC > 1) in uterine fluid samples from hUC-MSC/CS participants (*n* = 5) and controls (*n* = 6). (**C)** PCA scores plot to show 2 well-defined groups. Gene Ontology analysis in the biological process (**D**), cellular component (E), and molecular function terms (F) and Kyoto Encyclopedia of Genes and Genomes (KEGG) pathway analysis (G) for all 44 DEGs in the hUC-MSC/CS group. (H) The gene-pathway network was structured to identify key target genes. The proportion of endometrial cells (I) and immune cells (J) between hUC-MSC/CS participants and controls.

We performed bulk RNA-Seq analysis of endometrial fluid on the ET day in 5 patients from the hUC-MSC/CS group and 6 from the control group. A total of 44 DEGs, comprising 41 up- and 3 down-regulated genes in the hUC-MSC/CS group compared to the control group on the ET day (Figure 2A). Hierarchical clustering of these DEGs displayed 2 well-defined groups on the ET day (Figure 2B), and PCA further revealed distinct differences between the 2 groups (Figure 2C).

The 44 DEGs identified on the ET day were significantly enriched in immune-related processes, including T-cell activation under the biological process terms (Figure 2D), the external side of the plasma membrane in the cellular component terms (Figure 2E), and cytokine receptor binding in the molecular function terms (Figure 2F). KEGG pathway analysis indicated that genes in the hUC-MSC/CS group were mainly involved in cytokine-cytokine receptor interactions (Figure 2G). KEGG network analysis indicated that inhibin subunit beta A showed the most substantial change in the cytokine-cytokine receptor pathway (Figure 2H).

The deconvolution machine learning analysis revealed that the proportion of macrophages was higher in the hUC-MSC/CS group compared to the control group on the ET day, though the difference was not significant (Figure 2I). In contrast, the proportion of activated dendritic cells was significantly higher in the hUC-MSC/CS group than in the control group on the ET day (Figure 2J).

## Discussion

To the best of our knowledge, this is the first double-blind, RCT of hUC-MSCs seeded CS in treating RTE. In this trial, a trend towards a higher cLBR was observed in the hUC-MSC/CS group. Transcriptome analysis further revealed that hUC-MSCs may play an immunomodulatory role in embryo implantation.

Our data suggest that patients in the hUC-MSC/CS group are more likely to meet the ET criteria, with an increased EmT and improved EmP, leading to lower cycle cancelation rates and greater opportunities for ET. Of note, the number of cumulative embryos transferred was marginally higher in the hUC-MSC/CS group as compared to the controls. It is now generally accepted that human MSCs primarily function through paracrine signaling rather than engraftment into damaged tissues.^27-29^ The angiogenic potential of MSCs secretome has been confirmed,^30,31^ which may contribute to the endometrial lining reconstruction. Additionally, accumulating evidence indicates that human MSCs exert profound immunoregulatory effects through both cell-cell contact mechanisms and the activation of the inflammatory microenvironment.^32^ Comprehensive data mining of our RNA-seq data on the ET day also highlighted the immunomodulatory effects of hUC-MSCs on embryo implantation. We speculate that the numerically higher live birth rate in the hUC-MSC/CS group was achieved through the amelioration of impaired endometrial receptivity, rather than merely an improvement in EmT and EmP. In our previous study using a rat model of thin endometrium, we observed that the expression of endometrial receptivity markers, integrin αγβ3, and leukemia inhibitor factor, was significantly up-regulated in the BMDSCs transplantation group.^33^

Two out of 5 pregnancies ended up with early abortion in the hUC-MSC/CS group. The etiology of early spontaneous abortion is attributed to embryonic, maternal/parental, and environmental factors, with embryonic chromosomal abnormalities accounting for more than half of all risk factors.^34^ We performed genomic copy number variation sequencing on the miscarried tissue to rule out embryonic factors. Therefore, it was inferred that the incompetence of endometrium should be blamed for the miscarriages. However, the occurrence of early pregnancy indicates partial repair of the endometrium. Regarding safety concerns, the incidence of placenta accreta was significantly higher in the MSC group than in the control group, as confirmed by a previous RCT.^35^ Endometrial trauma and the mechanisms that control the physiological trophoblast cell migration contributes to placenta accreta.^36,37^ Previous studies have demonstrated that hUC-MSC-derived exosomes promote the proliferation, migration, and invasion of trophoblast cells.^38^ Thus, it was postulated that hUC-MSCs promote the occurrence of placenta accreta by enhancing the migration and invasion of trophoblast cells, under the premise of endometrial injury. No studies have yet reported the maternal outcomes, such as gestational hypertension, preeclampsia, etc. Our study was underpowered to detect significant differences in the aforementioned maternal outcomes. Larger RCTs with maternal and neonatal complications being the primary outcomes are needed to elucidate the association between MSCs treatment and maternal and neonatal outcomes.

AEs observed in our trial, including dizziness, nausea, and postoperative lower abdominal pain, were attributed to cervical dilatation rather than stem cell infusion. We observed that 2 out of 11 participants in the hUC-MSC/CS group developed urticaria, which might be treatment-related AEs. The triglyceride elevation after stem cell therapy observed in our trial was not consistent with previous research.^25^ We attributed this elevation to the high-dose estrogen used in HRT-FET cycles.^39^ We believe these findings suggest a paradigm shift in managing estrogen usage for FET cycles. Reducing the estrogen dosage or switching from a hormonal cycle to using a natural cycle may be viable alternatives after cell therapy.

Previous trials were well-designed, prospective, self-controlled studies assessing the efficacy and safety of stem cell treatment for recurrent AS or treatment-resistant thin endometrium.^5,14-16,40-42^In our trial, EmT improved significantly in the pre- and post-hUC-MSC/CS groups, while no statistically significant difference was observed in the control group, suggesting that farming therapy holds potential for endometrial regeneration. Unlike previous research designs, we conducted a prospective double-blind RCT. Recently, an RCT compared the effects of an autologous BMDSC-scaffold group versus a control group on the ongoing pregnancy rate in women with IUA.^35^ Most subjects in that trial were not diagnosed as RTE since more than 70% had not previously undergone hysteroscopic adhesiolysis. On the contrary, we focused on participants with RTE who had undergone an average of 4 to 5 adhesiolyses without achieving conception. Besides, we set saline/CS complex as the control group, while the previous RCT employed standard treatment (adhesiolysis combined with Foley balloon catheter) as the control.

The highlight of our treatment strategy was the use of a plowing technique on uterine scar areas prior to hUC-MSC/CS transplantation, based on the concept of “scarification and fertilization.”^8^ The initial stem cell niche was around vascularity,^43^ and the plowing technique was intended to enhance the integration of transplanted exogenous stem cells into this niche. We coined the term “farming therapy” for this combined procedures, wherein fibrous tissues were plowed first, followed by the transplantation of cell seeds to promote functional regeneration of the endometrium. Another strength of the study was the inclusion of mental health as a variable; participants in both groups tended to experience improved psychological well-being following the intervention.

There are some limitations in our study. The sample size was limited due to ethical considerations, making it appropriate to regard this research as a pilot study. Nevertheless, our results could inform power analysis estimates for future larger-scale RCTs. Moreover, further follow-up is necessary to assess the long-term safety of stem cell therapy for both participants and their offspring.

## Conclusions

In this double-blind, RCT involving patients with treatment-refractory thin endometrium, farming therapy using an hUC-MSCs-seeded scaffold resulted in a numerically higher cLBR than the control. Short-term follow-up over one year confirmed the relative safety of the hUC-MSC/CS complex. Moreover, hUC-MSCs could modulate the cytokine-cytokine receptor interaction pathway via paracrine signaling, potentially enhancing endometrial receptivity and facilitating embryo implantation. Therefore, this novel therapy can be proposed to patients with RTE.

## Supplementary Material

szaf011_suppl_Supplementary_Figures_S1-S4_Tables_S1-S6

## Data Availability

Data are available from the corresponding author on reasonable request.

## Notes

**TRIAL REGISTRY NAME:** ClinicalTrials.gov

**TRIAL REGISTRATION NUMBER:** NCT05495711.
