# Supplementary material for: hUC-MSCs loaded collagen scaffold for refractory thin endometrium caused by Asherman syndrome: a double-blind randomized controlled trial
Source: Stem Cells Transl Med. 2025 May 15;14(4):szaf011. doi: 10.1093/stcltm/szaf011 (PMC12079654; doi:10.1093/stcltm/szaf011)
Supplement: szaf011_suppl_Supplementary_Figures_S1-S4_Tables_S1-S6 [file szaf011_suppl_supplementary_figures_s1-s4_tables_s1-s6.pdf]

Table of contents

Item	Description	Page
Additional exclusion criteria	Additional exclusion criteria	2
Introduction of the deconvolution method	Introduction of the deconvolution method	3
Figure S1	Characterization of clinical-grade hUC-MSCs.	4
Figure S2	Gestational sacs obtained in the hUC-MSC/CS group.	6
Figure S3	Representative 3D ultrasound images of endometrium in the two groups.	7
Figure S4	Representative 2D ultrasound images of endometrium in the two groups.	8
Table S1	Suppliers of antibodies and differentiation kit.	9
Table S2	Definitions of secondary efficacy and safety outcomes.	10
Table S3	Pre- and post-mean score of SCL-90 between the two groups.	12
Table S4	Pre- and post-mean score of SCL-90 within each group.	13
Table S5	Adverse events and serious adverse events of the trial.	14
Table S6	Clinical characteristics of patients who got pregnant in hUC-MSC/CS group.	15

Additional exclusion criteria

1. uncured sexually transmitted diseases.
2. pregnant or participated in other clinical studies in recent 3 months, or any other reason that the researchers considered not to participate in the trial.
3. Coagulation disorders or other hematological disorders.
4. Abnormal uterine bleeding.
5. Hypertension, heart, kidney and liver insufficiency, urea nitrogen (BUN) or serum creatinine (Cr) > 2 times of normal upper limit, serum alanine aminotransferase (ALT) or aspartate aminotransferase (AST) > 2·5 times of normal upper limit.
6. Active genital tuberculosis.
7. Immune system diseases.

Introduction of the deconvolution method

The routine clinical adoption of single-cell RNA-seq (scRNA-seq) is hindered by the requirement for more intricate sample processing and higher costs. A practical alternative to scRNA-seq is the deconvolution method, which directly estimates the proportions of different cell types from bulk RNA-seq data[25,26]. This machine learning-based deconvolution approach leverages a pre-constructed cell type-specific feature matrix tailored to a particular tissue, allowing for the extraction of single-cell information from bulk tissue transcriptome data. It facilitates the identification and quantification of individual cell types within the tested bulk tissue, providing cell type-specific gene expression profiles. In this study, the cell-specific gene expression matrix represents 22 distinct immune cell subtypes, while a distinct matrix corresponds to gene expression data obtained from bulk sequencing of endometrial tissue. By employing this methodology, it is possible to accurately determine the relative proportions of these 22 cell subtypes.

Figure S1

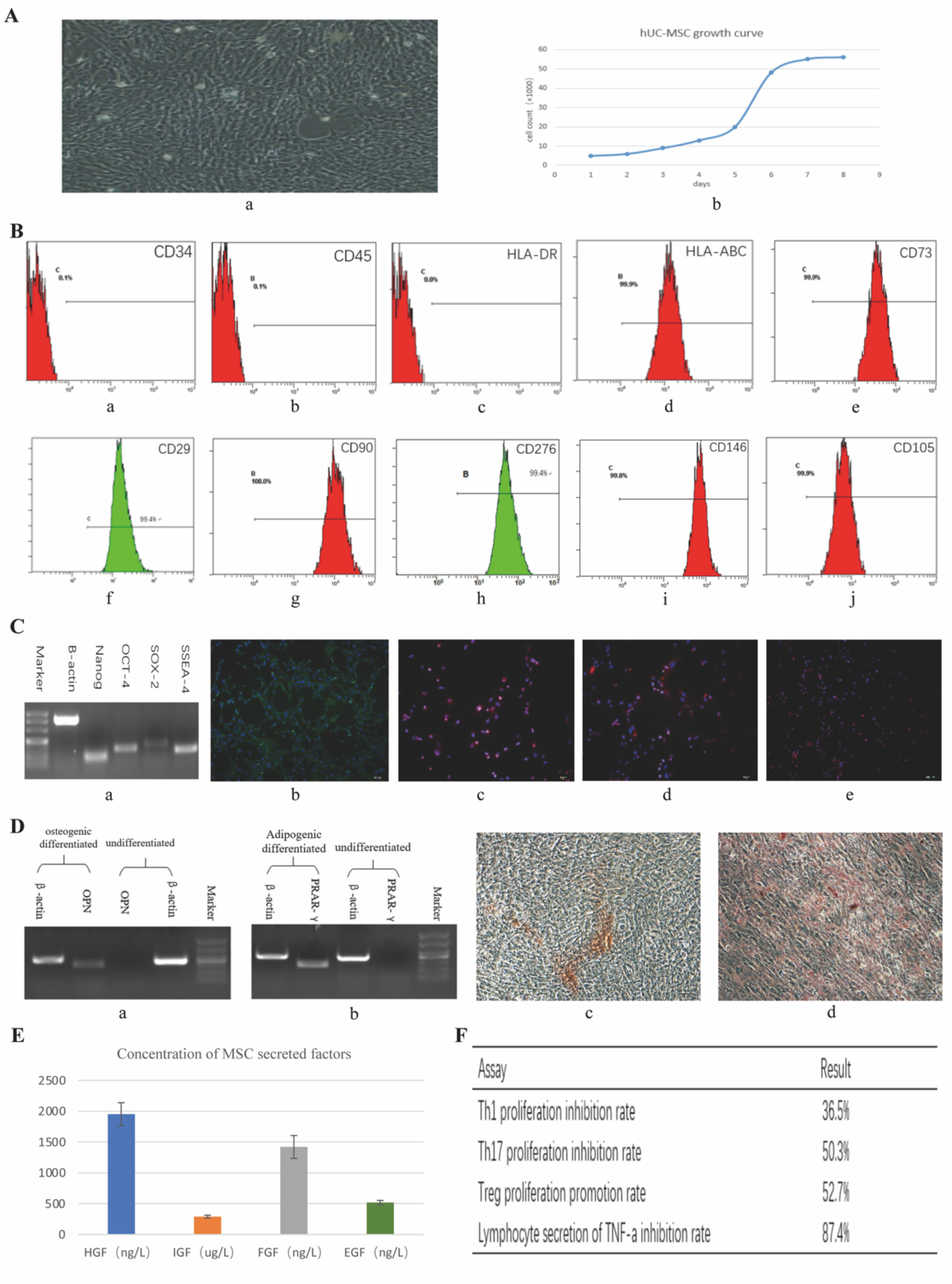

Figure S1. Characterization of clinical-grade hUC-MSCs.

(A.a) hUC-MSC morphology; (A.b) hUC-MSC growth curve; (B.a-B.j) cell surface marker of CD45, CD34, HLA-ABC, HLA-DR,CD73, CD105, CD29, CD90, CD276, and CD146, respectively (FACS); (C.a) SSEA-4, OCT-4, Nanog and SOX-2 (RT-PCR); (C.b-C.e) multipotent protein expression of SSEA-4 , OCT-4, Nanog and SOX-2 (immunofluorescence staining); (D.a-D.b) mRNA expression for PRAR-

r, OPN (before and after osteogenic and adipogenic differentiation); (D.c-D.d) Osteogenesis and adipogenesis under light microscope; (E) the concentration of MSC secreted factors; (F) Specific Lymphocyte Subpopulation Testing (co-culture of hUC-MSC with PBMC in 1:5 ratio).

Figure S2

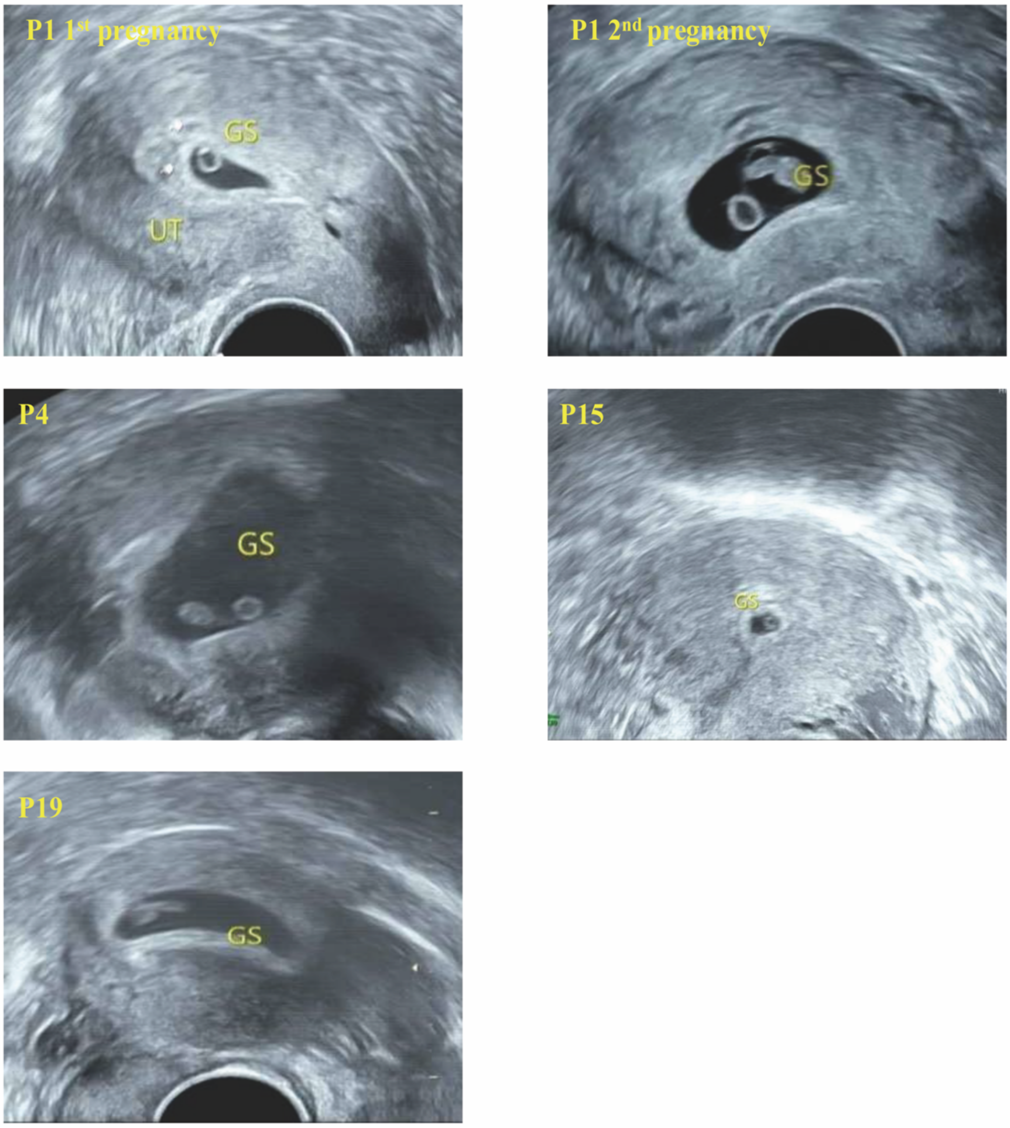

Figure S2. Gestational sacs obtained in the hUC-MSC/CS group.

Figure S3

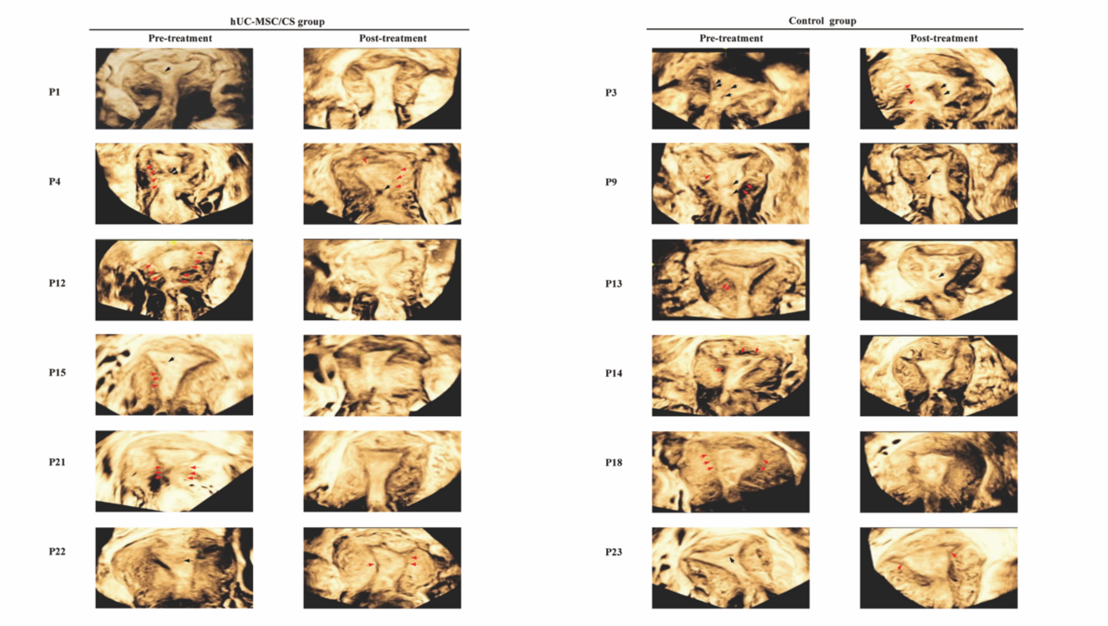

Figure S3. Representative 3D ultrasound images of endometrium in the two groups. The black arrows point to the damaged area of the endometrium. The red arrows point to the unclear borders or defects of the endometrium.

Figure S4

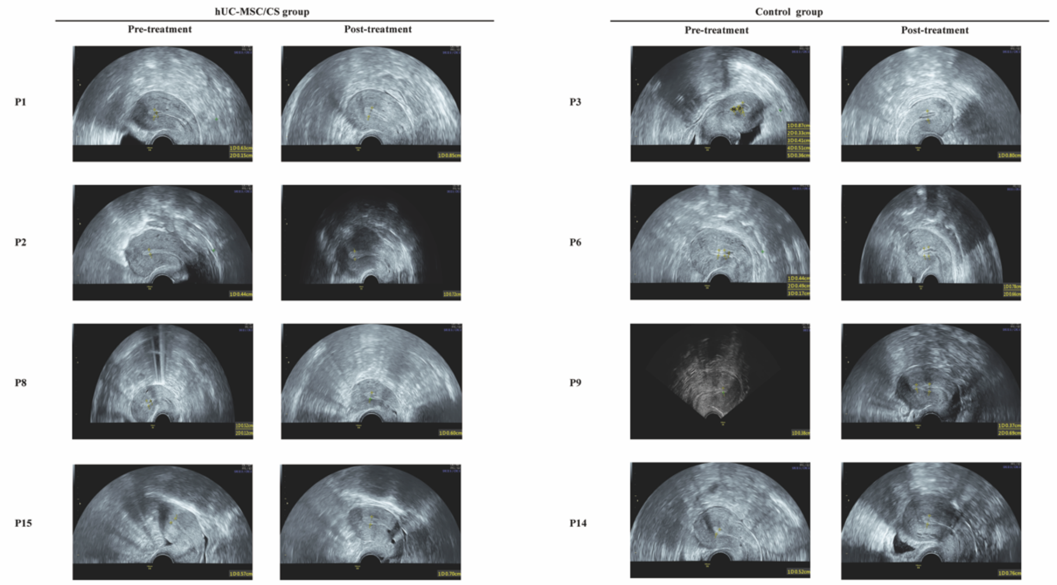

Figure S4. Representative 2D ultrasound images of endometrium in the two groups.

Table S1. Suppliers of antibodies and differentiation kit.

Reagents	Suppliers
Hu HLA-ABC PE DX17	BD Pharmingen, USA
Hu CD105 FITC 266	BD Pharmingen, USA
Hu CD29 PE TS2/16	BD Pharmingen, USA
Hu CD90 FITC 5E10	BD Pharmingen, USA
Hu CD276 BV421 7-517	BD Pharmingen, USA
Hu CD146 PE P1H12	BD Pharmingen, USA
Hu CD45 FITC HI30	BD Pharmingen, USA
Hu CD73 PE MAB	BD Pharmingen, USA
Hu HLA-DR FITC TU36	BD Pharmingen, USA
Hu CD34 FITC	BD Pharmingen, USA
hUC-MSC adipogenic differentiation kit	OriCell,China
hUC-MSC osteogenic differentiation kit	OriCell,China

Table S2. Definitions of secondary efficacy and safety outcomes.

Secondary outcome	Definition
Cumulative biochemical pregnancy	Biochemical pregnancy was defined as a serum concentration of β-hCG over 10 mIU/ml.
Cumulative clinical pregnancy	Clinical pregnancy was defined as gestational sac visible on ultrasound at approximately 6 weeks of gestation.
Implantation	Implantation was defined as the embryo that result in visible gestational sac at the stage of clinical pregnancy.
Cumulative ongoing pregnancy	Ongoing pregnancy was defined as a viable pregnancy at 12 weeks of gestation.
Cumulative early spontaneous abortion	Spontaneous abortion before 12 weeks of gestation.
Cumulative ectopic pregnancy	Ectopic pregnancy was defined as a visible gestational sac outside the endometrial lining of uterus cavity.
Endometrial thickness	The maximum distance from the anterior to posterior wall of endometrium-myometrium interfaces.
Endometrial pattern	The ultrasonic index reflecting endometrial proliferation and stromal decidualization which was divided into three types: A,B, and C.
Symptom Check List	The most widely used scale for mental disorders.
Gestational diabetes mellitus (GDM)	GDM was defined as carbohydrate intolerance of variable severity with onset or first recognition during pregnancy as determined from the diagnosis in the obstetrical medical record.
Preeclampsia	Preeclampsia was defined as the development of gestational hypertension with proteinuria (≥300mg/24-hour urine collection or 30mg/dL in single urine sample) of new onset after 20 weeks of gestation.
Gestational hypertension	Gestational hypertension was characterized by the development of blood pressure greater than 140/90 mmHg after pregnancy without proteinuria or other signs of preeclampsia.
Intrahepatic cholestasis of pregnancy (ICP)	a common disorder of pregnancy manifested by pruritus and elevated bile acids
Premature rupture of membrane (PROM)	PROM was defined as rupture of amniotic membrane before the onset of labor, including term PROM and preterm PROM.
Preterm delivery	Delivery of a baby at more than 28 and less than 37 weeks gestational age.

Placenta previa	Placenta previa was characterized by a placenta that is implanted over or very near the internal cervical os.
Placental abruption	Placental separation from its implantation site before delivery.
Placental adhesion disorder	Defined as invasion of chorionic villi into the myometrium.
Congenital anomalies	Congenital anomalies referred to structural or functional anomalies that occur during pregnancy, including minor and major anomalies.
Postpartum hemorrhage	Postpartum hemorrhage was defined as the loss of 500 ml of blood or more after completion of the third stage of labor.
Puerperal infection	Bacterial infection of the genital tract after delivery and during puerperium.
Stillbirth	The absence of signs of life at birth.
Neonatal respiratory distress syndrome	Respiratory insufficiency with hypoxemia and compensatory tachypnea for newborns.
Neonatal jaundice	Neonatal jaundice was a yellowing of skin and other tissues of a newborn infant.
Neonatal infection	A variety of infections in neonate caused by bacteria, virus, fungi, etc.
Neonatal death	The death of a live-born neonate within 28 days after delivery.

Table S3. Pre- and post-mean score of SCL-90 between the two groups.

Mean score	Pre-			Post-		
	hUC-MSC/CS	Control	P	hUC-MSC/CS	Control	P
Global Severity Index	1.2(1.1-1.4)	1.2(1.1-1.4)	0.42	1.1(1.0-1.4)	1.1(1.1-1.3)	0.87
Somatization	1.2±0.2	1.2±0.2	0.98	1.2±0.2	1.2±0.3	0.77
Obsessive-compulsive	1.3(1.1-1.7)	1.2(1.1-2.0)	0.78	1.2(1.1-1.5)	1.3(1.1-1.7)	0.87
Interpersonal sensitivity	1.1(1.0-1.4)	1.2(1.1-1.4)	0.73	1.0(1.0-1.3)	1.0(1.0-1.1)	0.46
Depression	1.2(1.0-1.3)	1.2(1.1-1.5)	0.39	1.2(1.0-1.3)	1.1(1.1-1.3)	0.91
Anxiety	1.1(1.0-1.2)	1.1(1.0-1.4)	0.53	1.1(1.0-1.3)	1.1(1.0-1.4)	0.73
Hostility	1.3(1.0-1.7)	1.2(1.0-1.3)	0.49	1.2(1.0-1.3)	1.2(1.0-1.3)	0.61
Phobic anxiety	1.0(1.0-1.1)	1.0(1.0-1.1)	0.82	1.0(1.0-1.0)	1.0(1.0-1.0)	0.53
Paranoid ideation	1.0(1.0-1.2)	1.2(1.0-1.3)	0.53	1.0(1.0-1.2)	1.0(1.0-1.3)	0.69
Psychoticism	1.1(1.0-1.3)	1.1(1.0-1.4)	0.87	1.0(1.0-1.3)	1.0(1.0-1.2)	0.69
Other	1.4(1.0-1.4)	1.3(1.1-1.4)	0.78	1.3(1.0-1.6)	1.1(1.0-1.4)	0.25

Table S4. Pre- and post-mean score of SCL-90 within each group.

Mean score	hUC-MSC/CS			Control		
	Pre-	Post-	P	Pre-	Post-	P
Global Severity Index	1.2(1.1-1.4)	1.1(1.0-1.4)	0.17	1.2(1.1-1.4)	1.1(1.1-1.3)	0.03
Somatization	1.2±0.2	1.2±0.2	0.90	1.2±0.2	1.2±0.3	0.43
Obsessive-compulsive	1.3(1.1-1.7)	1.2(1.1-1.5)	0.04	1.2(1.1-2.0)	1.3(1.1-1.7)	0.61
Interpersonal sensitivity	1.1(1.0-1.4)	1.0(1.0-1.3)	0.20	1.2(1.1-1.4)	1.0(1.0-1.1)	0.01
Depression	1.2(1.0-1.3)	1.2(1.0-1.3)	0.67	1.2(1.1-1.5)	1.1(1.1-1.3)	0.29
Anxiety	1.1(1.0-1.2)	1.1(1.0-1.3)	0.71	1.1(1.0-1.4)	1.1(1.0-1.4)	0.49
Hostility	1.3(1.0-1.7)	1.2(1.0-1.3)	0.22	1.2(1.0-1.3)	1.2(1.0-1.3)	0.11
Phobic anxiety	1.0(1.0-1.1)	1.0(1.0-1.0)	0.04	1.0(1.0-1.1)	1.0(1.0-1.0)	0.06
Paranoid ideation	1.0(1.0-1.2)	1.0(1.0-1.2)	0.58	1.2(1.0-1.3)	1.0(1.0-1.3)	1.0
Psychoticism	1.1(1.0-1.3)	1.0(1.0-1.3)	0.24	1.1(1.0-1.4)	1.0(1.0-1.2)	0.14
Other	1.4(1.0-1.4)	1.3(1.0-1.6)	0.75	1.3(1.1-1.4)	1.1(1.0-1.4)	0.07

Table S5. Adverse events and serious adverse events of the trial.

	hUC-MSC/CS<br>n=11	Control<br>n=13	P
Adverse events			
Fever-No.(%)	0 (0.0)	0 (0.0)	-
Chill-No.(%)	0 (0.0)	0 (0.0)	-
Dizziness-No.(%)	2 (18.2)	2 (15.4)	1.00
Somnolence-No.(%)	0 (0.0)	0 (0.0)	-
Headache-No.(%)	0 (0.0)	0 (0.0)	-
Nausea-No.(%)	2 (18.2)	2 (15.4)	1.00
Postoperative lower abdominal pain-No.(%)	5 (45.5)	7 (53.8)	1.00
Abnormal vaginal bleeding-No.(%)	0 (0.0)	0 (0.0)	-
Infection-No.(%)	0 (0.0)	0 (0.0)	-
Urticaria-No.(%)	2 (18.2)	0 (0.0)	-
Serious adverse events			
Uterus perforation-No.(%)	0 (0.0)	0 (0.0)	-
Fetus malformations-No.(%)	0 (0.0)	0 (0.0)	-

Table S6. Clinical characteristics of patients who got pregnant in hUC-MSC/CS group.

**Patient**	**Age (y)**	**Symptoms**	**Etiology**	**Prior repair attempts**	**Maximal IUA score**	**Previous treatment received**	**EmT (pre-/post-therapy mm)**	**Pregnancy outcome**
P01	29	Infertility (1 yr) Hypomenorrhea	2 D&C	3 HSA	10	Estrogen/ G-CSF/ traditional Chinese medicine	5.0/8.2	2 ET, spontaneous abortion at 7 weeks (1st ET) and natural labor at 39 weeks (2nd ET), boy, 2900g, Apgar 8/10
P04	34	Infertility (3 yrs) Hypomenorrhea	1 induced labor	4 HSA	10	Estrogen/ G-CSF/ Aspirin/ traditional Chinese medicine	6.4/7.3	3 ET and cesarean section at 38 weeks, girl, 3200g, Apgar 10/10
P15	36	Infertility (1 yr) Hypomenorrhea	1 D&C	3 HSA	10	Estrogen/ G-CSF/ Aspirin/ hyperbaric oxygen/ traditional Chinese medicine	5.2/6.8	3 ET and spontaneous abortion at 7 weeks
P19	33	Infertility (1 yr) Hypomenorrhea	1 D&C	8 HSA	12	Estrogen/ Aspirin/ traditional Chinese medicine	6.4/8.0	1 ET and cesarean section at 34 weeks, boy, 2350g, Apgar 10/10
